# Paraoxonase gene polymorphisms and haplotype analysis in a stroke population

**DOI:** 10.1186/1471-2350-7-28

**Published:** 2006-03-21

**Authors:** Alireza Pasdar, Helen Ross-Adams, Alastair Cumming, John Cheung, Lawrence Whalley, David St Clair, Mary-Joan MacLeod

**Affiliations:** 1 Department of Mental Health, University of Aberdeen, UK; 2 Department of Molecular and Cell Biology, University of Aberdeen, UK; 3 Department of Medicine and Therapeutics, University of Aberdeen, UK

## Abstract

**Background:**

Paraoxonase (*PON*) has anti-atherogenic activity due to its protective function against low density lipoprotein (LDL) oxidation. Alteration of enzyme activity due to polymorphisms in the *PON *genes may influence the development of atheroma and thus affect stroke risk. Three *PON *genes (*PON1*, *PON2 *and *PON3*) have been identifiedand mapped to chromosome 7.

**Methods:**

We looked at the distribution of paraoxonase polymorphisms and haplotype arrangement in 397 Caucasian ischaemic stroke patients and 405 controls. We investigated 6 different common single nucleotide polymorphisms (SNP) in *PON *genes; two substitutions in *PON1 *["A/G": Gln (Q)/Arg (R)] at codon 192 and ["T/A": Leu (L)/Met (M)] at codon 55, two in *PON2 *at codon 311 ["G/A": Cys (C)/Ser (S)] and codon 148 ["C/G": Ala (A)/Gly (G)] and two SNPs, both "A" to "G" substitutions, in *PON3 *– intronic rs2074353, which we designated *PON3*-1 and [Ala (A)/Ala (A)] at codon 99, designated as *PON3*-3. Dynamic Allele Specific Hybridisation (DASH) was used as the genotyping assay. Haplotype analysis was performed using both PHASE and EHPLUS programs.

**Results:**

Genotype and allele frequencies were similar in cases and controls. Lipid profiles were not influenced by *PON *genotype. Haplotype frequencies for the six loci (*PON2*-148, *PON2*-311, *PON3*-3, *PON3*-1, *PON1*-55 and *PON1*-192) were estimated. Comparison of the two programs showed a significant difference in haplotype arrangements with EHPLUS (p-value = 0.005) but not with PHASE Ver.2 (p-value = 0.12). The 112211 (1 = frequent allele, 2 = rare allele) haplotype arrangement was commoner in cases than controls (p = 0.015), and the 111121 haplotype was commoner in controls (p = 0.006).

**Conclusion:**

Our study did not identify a role for individual paraoxonase gene polymorphisms in the pathogenesis of ischaemic stroke. Findings of haplotype differences should be confirmed in large scale studies. The importance of using a well-validated haplotype analysis program is also underlined.

## Background

Ischaemic stroke is a complex trait, with genetic influences likely to work through various risk factors for cerebrovascular disease. Hypertension and dyslipidemia are known to influence atherosclerosis and thus predispose to ischaemic stroke. Although lipids are a weaker risk factor for stroke than blood pressure or age, hyperlipidemia contributes to stroke risk [1], while lipid lowering therapy significantly reduces the risk of stroke [2]. Low HDL has also been reported as a risk factor for ischaemic stroke [3]. Genes involved in lipid metabolism have been previously studied in the stroke population. Some studies suggest APOE*ε4 and APOE*ε2 alleles are associated with higher risk and lower risk of ischaemic stroke respectively, although this has not been confirmed in all studies [4,5].

Paraoxonase, a 45 kDa glycoprotein, is well known for its ability to detoxify organophosphates [6]. The process of atherosclerosis is thought to begin with inflammatory changes at the endothelial level, and oxidated LDL is believed to be pro-inflammatory. Oxidated lipids have also been associated with progression of carotid atherosclerosis [7,8]. As an HDL associated protein, paraoxonase protects LDL against oxidation [9], and thus may protect against atherosclerosis [10].

The paraoxonase gene maps to chromosome 7 (q21-23) and has three isoforms, *PON1*, *PON2 *and *PON3 *[11]. Some studies suggest *PON *polymorphisms might predispose to CHD or ischaemic stroke [12-14], while other studies are less conclusive [15]. *PON1 *has two common polymorphisms at codon 192 [A/G: Gln (Q)/Arg (R)] and 55 [T/A: Leu(L)/Met (M)]. These have been shown to correlate with enzyme activity [16,17]. Increased enzyme activity has been associated with higher HDL levels in some populations [18,19], but not in all studies [20]. The *PON 192 *and 55 polymorphisms have been associated with risk of ischaemic stroke in a small study of younger adults [13] and older patients respectively [21]. There are two common polymorphisms of the *PON2 *gene, *PON2*-148 [C/G: Ala (A)/Gly (G)] and *PON2*-311 [C/G: Cys (C)/Ser (S)], and the *PON2*-148 "G" (G variant) is associated with higher fasting blood glucose in NIDDM patients [22]. The *PON2*-311 "G" (S allele) has been associated with coronary artery diseases in different populations [23]. *PON3 *is thought to have a similar role to *PON1*[24].

The purpose of this study was to assess the distribution of polymorphisms of the *PON1*, *PON2 *and *PON3 *genes in well-phenotyped patients with ischaemic stroke and matched control subjects, and to expand on previous studies by examining haplotype distribution in these samples. We also examined the association between genotype and full lipid profile for a subgroup of controls.

## Methods

### Subjects

After ethical committee approval and informed consent, 397 Caucasian patients with a diagnosis of ischemic stroke were recruited over a period of 3 years. Four hundred and five Caucasian individuals from the same region, without any clinical cerebrovascular or overt cardiovascular disease and matched for age and sex were used as controls. For a subgroup of controls (n = 181) detailed lipid parameters including total cholesterol, VLDL-Cholesterol, VLDL Triglyceride, IDL, LDL, HDL2, HDL3 were available for comparison with *PON *genotypes.

On the basis of clinical, laboratory and radiological parameters, all stroke cases were classified according to Trial of Org 10172 in Acute Stroke Treatment (TOAST) criteria [25] to determine stroke subtype (Table 1).

**Table 1:** TOAST (Trial of Org 10172 in Acute Stroke Treatment) Classification of stroke patients

**Subtype**	**All Stroke Samples**	
	Number	%
Large Vessel Disease (LVD)	93	23.4%
Small Vessel Disease (SVD)	87	21.9%
Cardioembolic	99	25%
Other/Unclassified	118	29.7%
TOTAL	397	100%

### Markers and genotyping

DNA was extracted using Nucleon^® ^DNA extraction kits. 5–10 ng DNA was amplified through Hot Start PCR protocol using Immolase Taq DNA polymerase^®^. We used different sets of primers for each SNP as follows:

1-*PON1*-192 (A/G):

*PON1*-192F: 5'-biotin-CTATTTTCTTGACCCCTACTTA-3'

*PON1*-192R: 5'-CGCTAAACCCAAATACATCTCC-3'

2-*PON1*-55 (T/A):

*PON1*-55F: 5'-biotin-CTGACAGAAACTGGCTCTGAAG-3'

*PON1*-55R: 5'-AAGCCAGTCCATTAGGTAGTAT-3'

3-*PON2*-311 (C/G):

*PON2*-311F: 5'-biotin-GGTTCTCCGCATCCAGAACATT-3'

*PON2*-311R: 5'-AACTGTAGTCACTGTAGGCTTCTC-3'

4-*PON2*-148 (C/G):

*PON2*-148F: 5'-AGTGGAAATTTTTAAATTTGAAG-3'

*PON2*-148R: 5'-biotin-ACTGTTTTCAGATGCAACAGAGA-3'

5-*PON3*-rs2074353 (*PON3*-1, A/G):

*PON3*b-2074353F: 5'-biotin-TCTTCCTTTGTAGTGTGTTAGGAA-3'

*PON3*-2074353R: 5'-AATTTTTACATAACTCTTGAAACA-3'

6-*PON3*-rs2375003 (*PON3*-2, C/T):

*PON3*b-2375003F: 5'-biotin-CTGATCCCATGTAGATTAAATA-3'

*PON3*-2375003R: 5'-CGCTAGAAATCAGTGGTGGATT-3'

7-*PON3*-rs1053275 (*PON3*-3, A/G):

*PON3*b-1053275F: 5'-biotin-TGTCAAATCCACCACTGATTTCTA-3'

*PON3*-1053275R: 5'-GAACAAAACCCAAGGGCACAAG-3'

Of the three *PON3 *SNPs, only two SNPs [rs2074353 (*PON3*-1, A/G) and rs1053275 (*PON3*-3, A/G)] were confirmed in our population, with genotype frequencies in Hardy Weinberg equilibrium. All other SNPs were also in Hardy Weinberg equilibrium.

Genotyping assays for determining different alleles were based on the Dynamic Allele Specific Hybridisation (DASH) technique as described by the developers [26].

### Data analysis

Association between markers and disease phenotype was tested with Pearson χ^2 ^test. Multiple Logistic Regression was used to adjust for differences between samples in age, sex, smoking and hypertension. ANOVA was used to test for associations between lipid profiles and allelic distribution. Haplotype frequencies for the six loci (*PON2*-148, *PON2*-311 *PON3*-3, *PON3*-1, *PON1*-55 and *PON1*-192) were estimated for cases and controls, using and comparing two different validated programs: EHPLUS and PHASE Ver.2 [27,28].

## Results

Demographic parameters are shown in Table 2. Allele frequency for the six *PON *alleles investigated is shown in Table 3.

**Table 2:** Demographic parameters of cases and controls:

	**Cases (n = 397)**	**Controls (n = 405)**
Age (mean ± SD)	67.9 (± 11.5)*	67.2 (± 11.7)
Sex (M:F ratio)	1:1	1:1.3
Hypertension (%)	55.1	24.6
Total cholesterol (mmol/L) (mean ± SD)	5.6 (± 1.38)*	5.0 (± 2.0)
HDL (mmol/L) (mean ± SD)	1.21 (± 0.42)*	1.24 (± 0.6)
LDL (mmol/L) (mean ± SD)	3.65 (± 1.81)*	2.27 (± 1.49)
Triglycerides (mmol/L) (mean ± SD)	2.1 (± 1.6)*	1.9(± 1.1)
Atrial fibrillation	19.4%	-
Diabetes mellitus**	14.9%	3.2%
Previous IHD	33.5%	-
Carotid Stenosis (Out of 227 scanned, >50%, based on ACST criteria)	26%	-

* Non significant differences between cases and controls.

** History of diabetes or confirmed laboratory diagnosis.

**Table 3:** Allele Frequencies of 6 SNPs in *PON2*, *PON3 *&*PON1 *genes

	***PON2*-**		***PON3*-**		***PON1*-**	
	**148 A/G**	**311 C/G**	**3 A/G**	**1 A/G**	**55 T/A**	**192 A/G**
**Patients (n = 397)**	0.76/0.24	0.77/0.23	0.48/0.52	0.48/0.52	0.64/0.36	0.71/0.29
**Controls (n = 405)**	0.73/0.27	0.74/0.26	0.52/0.48	0.51/0.49	0.63/0.37	0.71/0.29
**P-value**	*0.29*	*0.16*	*0.11*	*0.20*	*0.73*	*0.89*
OR * (95% CI)	1.16 (0.88–1.52)	1.18 (0.94–1.48)	0.85 (0.69–1.04)	0.87 (0.71–1.07)	1.04 (0.84–1.27)	0.98 (0.79–1.22)

* Adjusted for age, sex and hypertension.

There was no significant difference between cases and controls by sex in genotype and allele frequencies. There was no significant difference in genotype and allele frequencies between cases and controls in terms of age stratification (≥65 and <65 years).

There was no significant difference in genotype or allele frequencies between cases and controls in the SVD group or LVD group, other than a small association between the GG genotype of *PON3 *and females with LVD (p = 0.03, OR = 2). *PON2*-311 "CC" genotype (SS variant) was higher in male cardioembolic (CE) stroke patients than in male controls (p = 0.03, OR = 2.05).

There was no association between lipid profile (LDL, HDL, total cholesterol/HDL ratio or triglycerides) and *PON *genotype in the stroke population. We also studied the association between *PON *genotype and full lipid profile in 181 of the controls. No differences were found in VLDL-Cholesterol, IDL, LDL, HDL2, HDL3, and total cholesterol concentrations in females or males between different genotypes of *PON1*-192, *PON1*-55, *PON2*-148 or *PON3*-1. VLDL-Triglyceride was higher in females with "GG" *PON3*-3 genotype (VLDL: AA genotype 69.8, "AG" genotype 77.8, "GG" genotype 116.0; p = 0.036).

Haplotype analysis was performed using PHASE version 2.02 and EHPLUS programs. The PHASE program [27], resulted in reconstruction of 16 different haplotypes in cases and controls. Overall haplotype frequencies in cases and controls were not significantly different (p = 0.19). However, some individual haplotypes were more frequent in one group than the other. Overall, the 112211 haplotype (1= frequent allele, 2= rare allele) was present at a higher frequency among cases than controls (16.62% vs 12.35%, p-value = 0.015, OR = 1.42 (1.07–1.87)). In contrast, 111121 was less frequent in cases than in controls (2.27% vs 4.81%, p = 0.006, OR = 0.46 (0.26–0.81)). There were also some differences in haplotype combination (genotype distribution) between cases and controls as follows: the "112211, 112221" haplotype (cases 10.6%, controls 5.2%: p = 0.005), the "111111, 112221" haplotype (cases 4%, controls 7.9%: p = 0.023).

We repeated analyses using EHPLUS [28] to compare haplotype arrangements in cases and controls. With this method, there was a significant difference (p = 0.005, df, 63) between groups. The most frequent haplotype in cases was 112211 and in controls 111111, while with PHASE2 the commonest haplotype for both groups was 112221. The EHPLUS program generated 64 different haplotypes both in cases and controls but only 32 had a frequency of more than 1%.

## Discussion

*PON1 *and *PON2 *are possible candidate genes for atherosclerotic diseases, especially coronary heart disease (CHD), but findings are inconsistent. In a recent meta-analysis of 43 studies [29], the 5 largest studies estimated the per-allele risk ratio at 1.05 for *PON1*-192 R (B) allele and combined analyses of studies of the *PON1*-55 M and *PON2*-311 C variants showed no significant overall associations with CHD. This differs from a recent study [30], not included in the meta-analysis, which suggested that the picture remains complicated. While there have been fewer studies in ischaemic stroke, there is some evidence of a role for the *PON1*-192 RR (BB) genotype and also for the *PON1 *(C-107T) allele in younger ischaemic stroke patients [13,14,31]. A recently published study of 81 stroke patients and 2053 controls from the CARE study also suggested a role for *PON1*-192 in predisposing to stroke, with no association with polymorphisms of the other *PON *genes. In contrast to this study, haplotype analysis or stroke subtyping was not performed [32]. There is evidence that paraoxonase (*PON1*) phenotype may predict vascular disease more accurately than genotype alone [33]. Due to logistical constraints in the current study it was not possible to measure *PON1 *phenotype, but future vascular studies should consider this.

As there are ethical difficulties in gaining consent from incapacitated individuals, this is not a study based on incident stroke. It is acknowledged therefore that patients with more severe stroke may not be included. However, the TOAST classification has similar distribution to other studies. The distribution of other vascular risk factors shows that these are high risk individuals. They form a subgroup of a Scottish stroke population which has been demonstrated to have an association with 5-lipoxygenase activating protein, an important mediator of the activity of cellular 5-Lipoxygenase (5-LO) which is a key enzyme in the biosynthesis of leukotrienes, and may thus be involved in atherosclerosis by proinflammatory mechanisms [34]. While a large number of individuals were unclassifiable by TOAST criteria, they all had CT evidence of ischaemic stroke. Individuals with cardioembolic stroke were also included. While these individuals may have a different underlying mechanism for their event, the role of oxidative stress through lipids driving atherothrombosis is likely to underlie the cardiac disease in most of these individuals.

Other than weak associations in small subgroups (females with LVD stroke, males with CE stroke), our study found no significance in overall allele or genotype frequency, although haplotype analysis suggests that the combination of certain alleles may increase or decrease the risk of ischaemic stroke in the whole population or in some specific subtypes. These results should be interpreted with caution due to the small numbers of individuals with specific haplotypes, but reinforces the need for very large scale studies to provide a definitive answer.

Although there are several programs based on different methods that can infer haplotypes, there is no consensus as to which method is the best [35]. Most programs, including EHPLUS, a modified version of EH developed by Ott, use the EM (Expectation-Maximisation) algorithm [28,35]. The program ignores all partial or missing genotype data so that the algorithm uses the full set of available data.

The PHASE program is based on coalescent models and uses Markov chain Monte Carlo methods [27,35]. These algorithms use a population genetics model to estimate different haplotype patterns, so a haplotype that is more similar to the commonly observed haplotype patterns is more likely to be inferred.

While the haplotype analysis is an important component of association studies in complex diseases [36], and the different computational methods have their individual strengths and weaknesses, significant haplotype differences in the absence of genotypic or allelic differences are difficult to interpret [35]. Our study highlights this further since one method (EHPLUS) suggests a statistically significant difference while PHSE fails to demonstrate an overall significant difference. It remains unclear which method is superior, but PHASE is believed to be more reliable for larger numbers of markers [35]. However, the level of significance is modest and could have arisen by chance.

In some genetic isolates certain *PON *genotypes can be associated with variation in plasma lipoproteins levels including LDL, HDL and total cholesterol [37-39]. In addition the physical relationship of *PON1 *with HDL and the existence of cholesterol pathway regulatory elements at the *PON1 *locus suggest a further relationship of *PON1 *with lipoproteins, which may contribute to its role of vascular diseases [40]. However, apart from a minor correlation between VLDL-Triglyceride and *PON3 *genotypes in female, we did not replicate these differences in our out-bred Scottish population.

## Conclusion

In conclusion, we found no significant genotypic or allelic frequency differences between stroke cases and controls for any of the structural polymorphisms of the *PON *genes tested. We identified specific haplotypes which may influence stroke risk in our population. Further work is required in large cohorts to determine whether these differences are real or have arisen by chance.

## Competing interests

The author(s) declare that they have no competing interests.

## Authors' contributions

AP participated in study design, carried out the genotyping and the statistical analyses and drafted the manuscript. HRA and JC helped with sample preparation LW and DS contributed to the study design, sample collection and helped draft the manuscript. AC helped with study design and commented on the manuscript. MJM contributed to study design, coordinated recruitment and drafted the manuscript. Authors read and approved the final manuscript.

## Pre-publication history

The pre-publication history for this paper can be accessed here:



## References

[B1] Gorelick PB, Schneck M, Berglund LF, Feinberg W, Goldstone J (1997). Status of lipids as a risk factor for stroke. Neuroepidemiology.

[B2] Collins R, Armitage J, Parish S, Sleigh P, Peto R, Group HPSC (2003). MRC/BHF Heart Protection Study of cholesterol-lowering with simvastatin in 5963 people with diabetes: a randomised placebo-controlled trial. Lancet.

[B3] Albucher JF, Ferrieres J, Ruidavets JB, Guiraud-Chaumeil B, Perret BP, Chollet F (2000). Serum lipids in young patients with ischaemic stroke: a case-control study. J Neurol Neurosurg Psychiatry.

[B4] Kessler C, Spitzer C, Stauske D, Mende S, Stadlmuller J, Walther R, Rettig R (1997). The Apolipoprotein E and beta-fibrinogen G/A-455 Gene Polymorphisms Are Associated With Ischemic Stroke Involving Large-Vessel Disease. Arterioscler Thromb Vasc Biol.

[B5] MacLeod MJ, De Lange RP, Breen G, Meiklejohn D, Lemmon H, Clair DS (2001). Lack of association between apolipoprotein E genoype and ischaemic stroke in a Scottish population. Eur J Clin Invest.

[B6] Costa LG, Li WF, Richter RJ, Shih DM, Lusis A, Furlong CE (1999). The role of paraoxonase (PON1) in the detoxication of organophosphates and its human polymorphism. Chem Biol Interact.

[B7] Mariani E, Polidori MC, Cherubini A, Mecocci P (2005). Oxidative stress in brain aging, neurodegenerative and vascular diseases: An overview. Journal of Chromatography B.

[B8] Salonen JT, Nyyssonen K, Salonen R, Porkkala-Sarataho E, Tuomainen TP, Diczfalusy U, Bjorkhem I (1997). Lipoprotein Oxidation and Progression of Carotid Atherosclerosis. Circulation.

[B9] Aviram M, Billecke S, Sorenson R, Bisgaier C, Newton R, Rosenblat M, Erogul J, Hsu C, Dunlop C, La Du B (1998). Paraoxonase Active Site Required for Protection Against LDL Oxidation Involves Its Free Sulfhydryl Group and Is Different From That Required for Its Arylesterase/Paraoxonase Activities : Selective Action of Human Paraoxonase Allozymes Q and R. Arterioscler Thromb Vasc Biol.

[B10] Salonen JT, Yla-Herttuala S, Yamamoto R, Butler S, Korpela H, Salonen R, Nyyssonen K, Palinski W, Witztum JL (1992). Autoantibody against oxidised LDL and progression of carotid atherosclerosis. Lancet.

[B11] Primo-Parmo SL, Sorenson RC, Teiber J, Du BNL (1996). The Human Serum Paraoxonase/Arylesterase Gene (PON1) Is One Member of a Multigene Family. Genomics.

[B12] Aubo C, Senti M, Marrugat J, Tomas M, Vila J, Sala J, Masia R (2000). Risk of myocardial infarction associated with Gln/Arg 192 polymorphism in the human paraoxonase gene and diabetes mellitus. Eur Heart J.

[B13] Voetsch B, Benke KS, Damasceno BP, Siqueira LH, Loscalzo J (2002). Paraoxonase 192 Gln->Arg Polymorphism: An Independent Risk Factor for Nonfatal Arterial Ischemic Stroke Among Young Adults. Stroke.

[B14] Voetsch B, Benke KS, Panhuysen CI, Damasceno BP, Loscalzo J (2004). The Combined Effect of Paraoxonase Promoter and Coding Region Polymorphisms on the Risk of Arterial Ischemic Stroke Among Young Adults. Arch Neurol.

[B15] Cao H, Girard-Globa A, Serusclat A, Bernard S, Bondon P, Picard S, Berthezene F, Moulin P (1998). Lack of association between carotid intima-media thickness and paraoxonase gene polymorphism in non-insulin dependent diabetes mellitus. Atherosclerosis.

[B16] Humbert R, Adler DA, Disteche CM, Hassett C, Omiecinski CJ, Furlong CE (1993). The molecular basis of the human serum paraoxonase activity polymorphism. Nat Genet.

[B17] Mackness B, Durrington PN, Mackness MI (1998). Human Serum Paraoxonase. Gen Pharmacol.

[B18] Obata T, Ito T, Yonemura A, Ayaori M, Nakamura H, Ohsuzu F (2003). R192Q paraoxonase gene variant is associated with a change in HDL-cholesterol level during dietary caloric restriction in nondiabetic healthy males. J Atheroscler Thromb.

[B19] van Himbergen TM, Roest M, de Graaf J, Jansen EHJM, Hattori H, Kastelein JJP, Voorbij HAM, Stalenhoef AFH, van Tits LJH (2005). Indications that paraoxonase-1 contributes to plasma high density lipoprotein levels in familial hypercholesterolemia. Journal of Lipid Research.

[B20] Nevin DN, Zambon A, Furlong CE, Richter RJ, Humbert R, Hokanson JE, Brunzell JD (1996). Paraoxonase Genotypes, Lipoprotein Lipase Activity, and HDL. Arterioscler Thromb Vasc Biol.

[B21] Schmidt R, Schmidt H, Fazekas F, Kapeller P, Roob G, Lechner A, Kostner GM, Hartung HP (2000). MRI Cerebral White Matter Lesions and Paraoxonase PON1 Polymorphisms : Three-Year Follow-Up of the Austrian Stroke Prevention Study. Arterioscler Thromb Vasc Biol.

[B22] Hegele RA, Connelly PW, Scherer SW, Hanley AJG, Harris SB, Tsui LC, Zinman B (1997). Paraoxonase-2 gene (PON2) G148 variant associated with elevated fasting plasma glucose in noninsulin-dependent diabetes mellitus. J Clin Endocrinol Metab.

[B23] Wang X, Fan Z, Huang J, Su S, Yu Q, Zhao J, Hui R, Yao Z, Shen Y, Qiang B, Gu D (2003). Extensive Association Analysis Between Polymorphisms of PON Gene Cluster With Coronary Heart Disease in Chinese Han Population. Arterioscler Thromb Vasc Biol.

[B24] Reddy ST, Wadleigh DJ, Grijalva V, Ng C, Hama S, Gangopadhyay A, Shih DM, Lusis AJ, Navab M, Fogelman AM (2001). Human Paraoxonase-3 Is an HDL-Associated Enzyme With Biological Activity Similar to Paraoxonase-1 Protein but Is Not Regulated by Oxidized Lipids. Arterioscler Thromb Vasc Biol.

[B25] Adams HPJ, Bendixen BH, Kappelle LJ, Biller J, Love BB, Gordon DL, Marsh EEIII (1993). Classification of subtype of acute ischemic stroke. Definitions for use in a multicenter clinical trial. TOAST. Trial of Org 10172 in Acute Stroke Treatment. Stroke.

[B26] Prince JA, Feuk L, Howell WM, Jobs M, Emahazion T, Blennow K, Brookes AJ (2001). Robust and Accurate Single Nucleotide Polymorphism Genotyping by Dynamic Allele-Specific Hybridization (DASH): Design Criteria and Assay Validation. Genome Res.

[B27] Stephens M, Smith NJ, Donnelly P (2001). A new statistical method for haplotype reconstruction from population data. Am J Hum Genet.

[B28] Zhao JH, Curtis D, Sham PC (2000). Model-Free Analysis and Permutation Tests for Allelic Associations. Hum Hered.

[B29] Wheeler JG, Keavney BD, Watkins PH, Collins PR, Danesh PJ (2004). Four paraoxonase gene polymorphisms in 11212 cases of coronary heart disease and 12786 controls: meta-analysis of 43 studies. Lancet.

[B30] Robertson KS, Hawe E, Miller GJ, Talmud PJ, Humphries SE (2003). Human paraoxonase gene cluster polymorphisms as predictors of coronary heart disease risk in the prospective Northwick Park Heart Study II. Biochim Biophys Acta.

[B31] Voetsch B, Damasceno BP, Annichino-Bizzacchi JM, Arruda VR, Loscalzo J (2001). Paraoxonase Polymorphisms and the Risk of Arterial Ischemic Stroke among Young Adults. Abstracts of the International Stroke Conference.

[B32] Ranade K, Kirchgessner TG, Iakoubova OA, Devlin JJ, DelMonte T, Vishnupad P, Hui L, Tsuchihashi Z, Sacks FM, Sabatine MS, Braunwald E, White TJ, Shaw PM, Dracopoli NC (2005). Evaluation of the Paraoxonases as Candidate Genes for Stroke: Gln192Arg Polymorphism in the Paraoxonase 1 Gene Is Associated With Increased Risk of Stroke. Stroke.

[B33] Jarvik GP, Rozek LS, Brophy VH, Hatsukami TS, Richter RJ, Schellenberg GD, Furlong CE (2000). Paraoxonase (PON1) Phenotype Is a Better Predictor of Vascular Disease Than Is PON1192 or PON155 Genotype. Arterioscler Thromb Vasc Biol.

[B34] Helgadottir A, Gretarsdottir S, St.Clair D, Manolescu A, Cheung J, Thorleifsson G, Pasdar A, Gran SF, Whalley LJ, Hakonarson HK, Thorsteinsdottir U, Kong A GJ, Stefansson K, MacLeod MJ (2005). Association of the Gene Encoding 5-Lipoxygenase Activating Protein to Stroke Replicated in a Scottish Population.. Am J Hum Genet.

[B35] Zhao H, Pfeiffer R, Gail MH (2003). Haplotype analysis in population genetics and association studies. Pharmacogenomics.

[B36] Yagil Y, Yagil C (2004). Candidate genes, association studies and haplotype analysis in the search for the genetic basis of hypertension. J Hypertens.

[B37] Boright AP, Connelly PW, Brunt JH, Scherer SW, Tsui LC, Hegele RA (1998). Genetic variation in paraoxonase-1 and paraoxonase-2 is associated with variation in plasma lipoproteins in Alberta Hutterites. Atherosclerosis.

[B38] Fanella S, Harris SB, Young TK, Hanley AJ, Zinman B, Connelly PW, Hegele RA (2000). Association between PON1 L/M55 polymorphism and plasma lipoproteins in two Canadian aboriginal populations. Clin Chem Lab Med.

[B39] Hegele RA, Brunt JH, Connelly PW (1995). A Polymorphism of the Paraoxonase Gene Associated With Variation in Plasma Lipoproteins in a Genetic Isolate. Arterioscler Thromb Vasc Biol.

[B40] Deakin S, Leviev I, Guernier S, James RW (2003). Simvastatin Modulates Expression of the PON1 Gene and Increases Serum Paraoxonase: A Role for Sterol Regulatory Element-Binding Protein-2. Arterioscler Thromb Vasc Biol.

